# Beyond the topics: how deep learning can improve the discriminability of probabilistic topic modelling

**DOI:** 10.7717/peerj-cs.252

**Published:** 2020-01-27

**Authors:** Noura Al Moubayed, Stephen McGough, Bashar Awwad Shiekh Hasan

**Affiliations:** 1 Department of Computer Science, Durham University, Durham, UK; 2 Department of Computer Science, University of Newcastle upon Tyne, Newcastle, UK; 3 Caspian Learning, Newcastle upon Tyne, Newcastle, UK

**Keywords:** Topic modelling, Stacked denoising autoencoders, Text classification, Sentiment analysis

## Abstract

The article presents a discriminative approach to complement the unsupervised probabilistic nature of topic modelling. The framework transforms the probabilities of the topics per document into class-dependent deep learning models that extract highly discriminatory features suitable for classification. The framework is then used for sentiment analysis with minimum feature engineering. The approach transforms the sentiment analysis problem from the word/document domain to the topics domain making it more robust to noise and incorporating complex contextual information that are not represented otherwise. A stacked denoising autoencoder (SDA) is then used to model the complex relationship among the topics per sentiment with minimum assumptions. To achieve this, a distinct topic model and SDA per sentiment polarity is built with an additional decision layer for classification. The framework is tested on a comprehensive collection of benchmark datasets that vary in sample size, class bias and classification task. A significant improvement to the state of the art is achieved without the need for a sentiment lexica or over-engineered features. A further analysis is carried out to explain the observed improvement in accuracy.

## Introduction

The rise of social media and online reviews has resulted in an exponential increase in the available data. Facebook has over a billion and a half active users a month (Smith, 2019) with billions of comments, and social reactions (in form of like, love, etc.). Twitter, the largest micro-blogging website, has 313 millions of users (Clement, 2019) writing 500 million tweets on a daily basis. Customer reviews are a standard feature of almost every online purchasing service (e.g. Amazon or Expedia). This vast wealth of data is raising the need for sophisticated analysis methods to better understand and exploit the knowledge hidden in the data.

A successful approach is probabilistic topic modelling, which follows a hierarchal mixture model methodology to unravel the underlying patterns of words embedded in large collections of documents (Blei, Carin & Dunson, 2010; Hofmann, 1999; Canini, Shi & Griffiths, 2009). The discovery of these patterns, known as topics, opens the doors for deeper analysis of the data including: clustering, sorting, summarisation, and prediction (Blei, Carin & Dunson, 2010).

Latent Dirichlet Allocation (LDA) (Blei, Ng & Jordan, 2003) is one of the most commonly used probabilistic topic modelling methods. It decomposes a collection of documents into its salient topics. A topic in LDA is a probability distribution over the documents’ vocabulary. LDA assumes a fixed number of topics set apriori and that each document may contain a combination of topics. LDA, and its variants (Teh et al., 2005; Porteous et al., 2008), is a completely unsupervised method with very few prior assumptions which has led to its popularity in text summarisation and clustering of large unstructured datasets. However, when labelled data is available it would be beneficial to include the class label in the model itself as demonstrated in (Mcauliffe & Blei, 2008; Perotte et al., 2011; Huh & Fienberg, 2012; Zhu, Ahmed & Xing, 2009).

Latent Dirichlet Allocation was customised to accommodate specific application area like sentiment analysis. Sentiment analysis tries to understand the sentiment behind the written text, for example product reviews. This problem has drawn a lot of attention in the last few years given the social and commercial impact it has (Bradley & Lang, 1999; Bravo-Marquez, Mendoza & Poblete, 2013; Go, Bhayani & Huang, 2009; Liu, 2012). In a highly competitive online market the deeper the understanding of customer views and attitudes the further advantage a business can have against the competition. Jo & Oh (2011) extended LDA to include sentence modelling with aspect/sentiment. Mei et al. (2007) and Lin & He (2009) explicitly model the sentiments in the data and then train the model in an unsupervised approach similar to the standard LDA. Topic modelling for sentiment analysis reduces the need for hand-crafted features and especially annotated corpora (Bradley & Lang, 1999; Cambria, Havasi & Hussain, 2012; Esuli & Sebastiani, 2006; Nielsen, 2011).

In this work, we take a novel approach to expand the modelling power of LDA within a supervised framework. The framework builds a separate LDA model per class. Each LDA is then used as a feature extractor to train a Stacked denoising autoencoder (SDA). The trained SDAs are used to generate the input to a simple classifier. The added layer of SDAs help further increase the disrciminability among the classes and hence achieve higher classification accuracy.

The introduced framework addresses the following points: (I) It avoids language specific sentiment lexicon and directly engineered features on the word/sentence level. Instead, we focus on modelling higher level abstract concepts/topics. (II) The system learns through hierarchical structure modelling to better understand the inter-dependencies among words and topics to convey the sentiment. (III) The framework is very general and can easily be adapted to different tasks and data sets with minimum feature engineering.

This approach is motivated by three key points:

- Context embedding: topic modelling through a probabilistic mixture approach (e.g. Latent Dirichilet Allocation) is highly advantageous in modelling the context in which words may appear given a sentiment. This also transfers the classification problem from the words space (or an engineered feature space of words) to the topic space making the whole system much more robust to noise.
- Structural modelling of topics: the use of SDA through a hierarchal structure of deep neural network captures, with minimum assumptions, the structural inter-dependencies among topics within the context of a sentiment (e.g. polarity, subjectivity, etc.).
- Simplified classification: as we will show in the methods and experiments, the combined use of topic modelling and SDA with our novel utilisation of reconstruction error (RE) results in highly separable feature space requiring only a simple linear classifier.

*The main contributions* of this work are:

- Develop a framework for automatic text classification with minimum feature engineering.
- Expand topic modelling by introducing an additional layer of abstraction to model the inter-relations amongst topics of the same text category.
- Introduce the RE of an auto-encoder as a surrogate measure of sample representation by the auto-encoder. The REs of the built SDAs are used as features for classification.
- Discriminability analysis to explain the benefit of SDAs and RE in enhancing the performance of the text classification task.

The framework is tested on ten benchmark datasets that vary in classification task, size, and domain with results significantly outperforming the state of the art on 8/10 of the datasets.

The next section reviews the related work in sentiment analysis. “Methods” details the methods used, followed by a description of the datasets used and the experimental design in “Data Sets and Experiments”. The results are reported in “Results”, while “Discussion” further examines the approach and the achieved results, and “Conclusion” summarizes the article.

## Related Work

Text classification problems are usually viewed as two tier problems: feature extraction and classification. Reviewing the state of the art in text classification is beyond the scope of this paper, however as we take sentiment analysis as an application we will briefly review the literature in this application.

### Feature extraction/engineering

Early work on sentiment analysis approached the problem from the traditional topic-based categorisation point of view. This involved standard natural language processing (NLP) techniques such as bag of words (Pang, Lee & Vaithyanathan, 2002), word vectors (Maas et al., 2011; Pouransari & Ghili, 2014), n-grams (Nguyen et al., 2014), and rule-based classifiers (Riloff, Wiebe & Phillips, 2005). Despite their initial success these methods performed worse than expected in comparison with other topic categorisation problems, as the approaches taken were not designed specifically for sentiment classification.

To incorporate prior information into the feature extraction method a lexical resource for sentiment analysis is needed to assign a polarity (e.g. positive, negative, or neutral) to the words independent of the context. Wilson, Wiebe & Hoffmann (2005) built the Opinion Finder lexicon on individual words and then used it to define sentiment at the level of phrases. A similar approach was taken by Zirn et al. (2011) which uses a sophisticated Markov chain method to predict the contextual sentiment of words within phrases or short text. Another commonly used lexicon for English language was presented by Bradley & Lang (1999) and later used for twitter sentiment analysis by Nielsen (2011). Other well-known lexicons include SentiStrength (Thelwall, Buckley & Paltoglou, 2012) and NRC (Mohammad & Turney, 2013). For more discussion and comparisons among the lexical resources the interested reader is referred to the work by Bravo-Marquez, Mendoza & Poblete (2013), where the authors also combined features from several lexical resources to enhance the performance of the overall system.

An appraisal group of sentiments was developed by Whitelaw, Garg & Argamon (2005) and then used to produce bag of words features. Based on the psychological definition of emotional states, Mohammad & Turney (2013) labelled a word bank for sentiment analysis. To establish more context aware features Kennedy & Inkpen (2006) used contextual valence shifters to rank words within a text which can then be fed to a classifier. Nguyen et al. (2014) used ratings (labelled or predicted) to enhance the performance of a word vector based sentiment analysis system. A sophisticated feature engineering approach was used for twitter data by Agarwal et al. (2011). It starts by using a polarity dictionary to assign a prior polarity to each word and then a tree representation of tweets is designed to combine many categories of features in one structure. A convolution kernel is then employed to compare among several trees. Tang et al. (2014b) used neural networks to learn sentiment specific word embedding (SSWE) to transform words into a continuous representation that takes into account the sentiment and syntactic context of words.

In the context of information retrieval, Eguchi & Lavrenko (2006) used a generative probabilistic model for topic modelling in order to retrieve documents with certain sentiment/polarity. The model extends the definition of topics within the text to model sentiments. The user would then request documents by topic and sentiment. A topic sentiment mixture model was proposed by Mei et al. (2007) which builds sentiment models for positive and negative opinions and extract topic models with their relevant sentiment coverage. Similarly a joint sentiment/topic model (JST) was presented by Lin & He (2009) that extends the well known unsupervised model, LDA. JST was further extended by Jo & Oh (2011) to handle words from several languages.

The advantage of topic modelling in general is that: (I) it allows for the extraction of useful information without the need for significant feature engineering. (II) The dimensionality of the resulting feature space can be set a-priori and is usually much smaller than the sparse feature vectors resulted from bag of words or n-grams. (III) LDA, and other Bayesian topic models, is adaptive by definition providing the overall system with the ability to handle streams of data very efficiently. (IV) transforming the input space to topics space makes the classifier less sensitive to noise. In “Methods” we present LDA in its original form before using it later to extract topic features (without modelling the sentiment). The sentiment modelling is carried out on a later stage using deep neural networks.

### Classification

Following topic categorisation problems several machine learning classification methods (mostly supervised) are commonly employed. Maximum Entropy classifier, which measures the amount of information the features can ‘tell’ about the polarity/sentiment, was used repeatedly (Pang, Lee & Vaithyanathan, 2002; Saif et al., 2013). Naive Bayes classifier was also used by Wu & Pao (2012). However, Support Vector Machines (SVM) are arguably the most widely used approach for sentiment prediction (Agarwal et al., 2011; Bravo-Marquez, Mendoza & Poblete, 2013; Nguyen et al., 2014; Wu & Pao, 2012).

More recently deep neural networks have been adopted for sentiment analysis after their impressive performance in several tasks under the umbrella of NLP (Collobert et al., 2011). Pouransari & Ghili (2014) utilised a recursive neural network (RNN) to model not only the individual words but how they appear in relationship to each other within a phrase of a given sentiment. RNN is commonly used in NLP due to their ability to model such structures. However, to represent complex relationships (e.g. negated positive) Socher et al. (2013) presented recursive neural tensor networks. A combined RNN model that takes into account the aspect extraction and sentiment representation was presented in Lakkaraju, Socher & Manning (2014) using a hierarchical deep learning framework. A dynamic convolutional neural network was used by Kalchbrenner, Grefenstette & Blunsom (2014) to handle input sentences of varying length capturing short and long-range relations, which could be particularly important for sentiment analysis. Tang et al. (2014a) used the same neural networks for SSWE but with added convolutional layers for category prediction. Wu & Pao (2012) built a deep feed-forward neural network to extract and classify high level features obtained from n-grams with the aim of reducing the complexity of feature engineering. A brief review and comparison of performance in sentiment analysis among RNN, and convolutional networks is discussed by Shirani-Mehr (2014) where the authors find, based on testing on one dataset only, that convolutional neural networks with word vector features performed better than the other networks or the baseline Naive Bayesian classifier.

Most relevant to our work are semi-supervised recursive autoencoders (AE) which were introduced for sentiment analysis by Socher et al. (2011). The authors used AE (described in “Stacked Denoising Autoencoders”) without a predefined structure with a combined reconstruction and cross entropy errors as the optimisation objective of the structural learning approach. AE based algorithms were used by Mirowski, Ranzato & LeCun (2010) to model a bag of words for text classification, topic modelling, and sentiment analysis tasks using a similar semi-supervised approach. Pollack (1990) introduced recursive AE as a compact distributed representation method for data, including textual data. However, the approach relied on binary word representation requiring a large sparse input space. To enhance generalisation a linear modification was introduced by Voegtlin & Dominey (2005) but with the same binary features. Word vector features were used by Socher et al. (2011) which are argued to be more suitable for AE, which require continuous data by definition.

## Methods

Our framework aims at finding a uniform way of representing variable-sized phrases and then using this representation effectively to achieve accurate sentiment classification. Topic modelling is used as a feature extraction method which provides a robust representation that requires minimum feature engineering independent of the language used and without the need for task-specific lexicon. It also converts a variable length document to a vector of probabilities (i.e. continuous variables) which has an advantage when modelling with stacked AE. AE uses the input topic model representation of all the documents labelled as conveying a specific task (e.g. sentiment) to build a structural representation that defines that task. REs, of the different AEs are then used by a simple classifier (we explore several options in “Classification Approach”) to provide the final prediction of the task perceived from the input document. Shifting the problem from word and phrases representation to topic representation has the advantage of building more dynamic and robust systems where small changes on the document level (or the introduction of new documents) will not cause a major change in how the topics are represented opening the door for adaptive text classification models that are able to cope with the fast changing content of the web.

**Figure 1:**
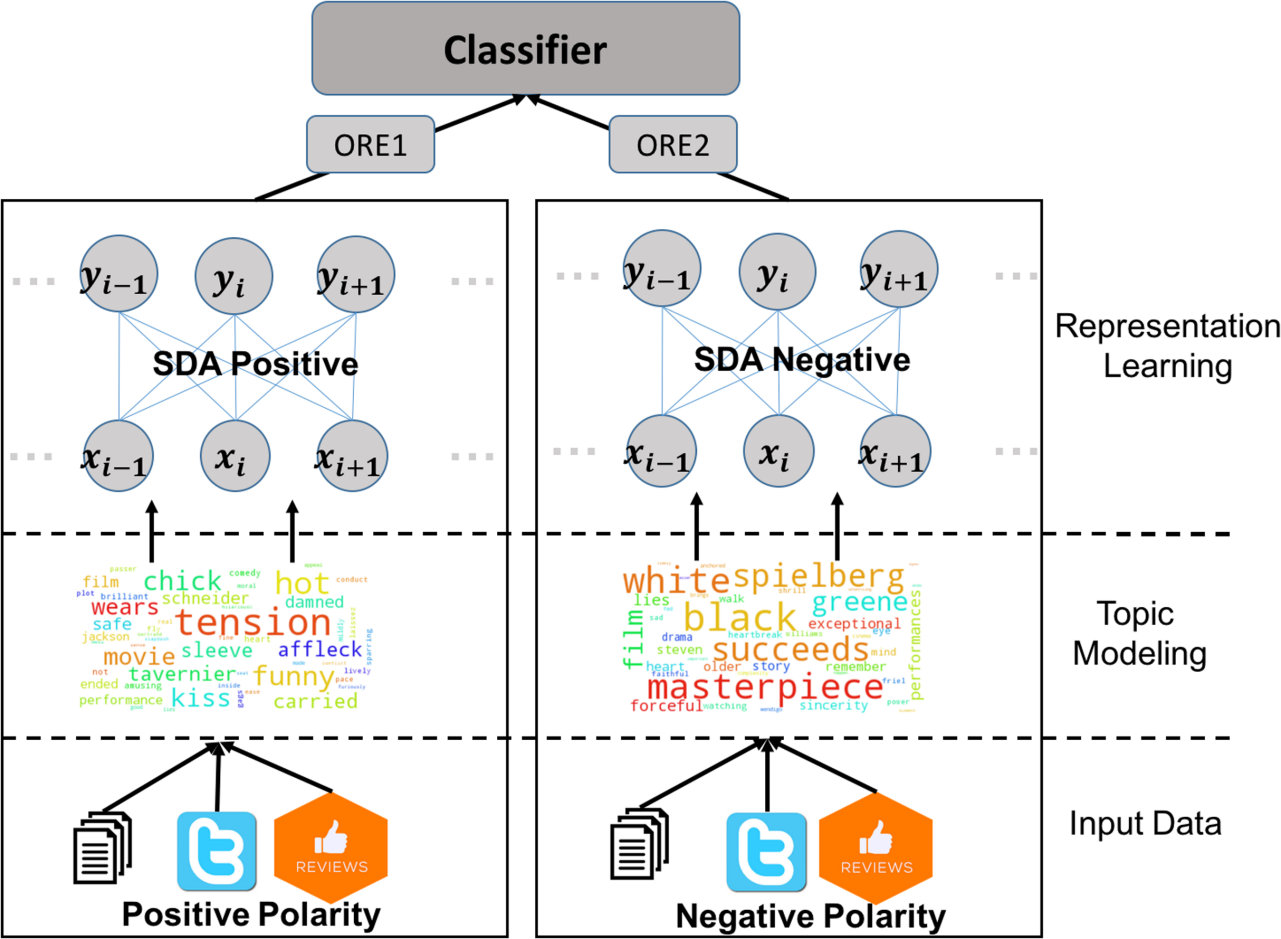
A schematic description of the overall classification scheme for sentiment analysis using the proposed framework. From bottom up the data come from various resources. Data is separated per sentiment and a topic model per sentiment is built. All the data are then passed through the topic models to generate features used by two SDAs for the positive and negative sentiment. Finally a classifier is used to predict the output.

Figure 1 describes the process of sentiment analysis using the proposed framework in this article. The input data of several resources is separated to negative and positive polarity. Two topic models are then built using the polarity specific data. The extracted features from each topic model are used to train the corresponding SDA model. To predict the sentiment of a given text, it is passed through the two topic models and the two SDAs resulting in an overall reconstruction error (ORE) per SDA which are used by a linear classifier to predict the polarity.

### Topic modeling

Consider, conceptually, that a phrase expressing a given task/sentiment is formed from a collection of words which are commonly used to express that sentiment. By capturing those task-related words, in the form of a topic, we should be able to capture the sentiment of a phrase (or document) more accurately than conventional keyword analysis. Through the building of a text/language model that transforms the words into an abstract (vector) representation should result in more informative features that are less affected by ‘noise’ at the word or even document level. This area of research is referred to as topic modelling (Blei, 2012).

Within the context of a given text classification task, topic models are able to automatically include contextual information, which is particularly helpful in cases where a word might reflect different sentiments in different domains/topics (e.g. ‘easy’ would be a positive sentiment in the context of the use of household items, but an ‘easy’ online game would be perceived negatively). Topic models are built as a Bayesian network (Blei, 2012) where the observed variables (the words) can be generated by realisation of random variables within the network. The network is equivalent to a mixture of models, that is a document is associated with the probability of it containing a topic and a document could include more than one topic. A word can be in more than one topic and a topic consists of more than one word. The probability of each document containing each of the topics can be used for further analysis.

Latent Dirichlet Allocation (Blei, Ng & Jordan, 2003) is the most commonly used method for topic modelling (Al Moubayed, Wall & McGough, 2017). LDA works by measuring the co-occurrence statistics of words/terms in a set of documents leading to recognising the topic structure within those documents. The only assumption made by LDA is the number of underlying topics, *k*, responsible for generating the documents, and a multinomial distribution of the topic over the words in the vocabulary. A document can then be seen to be generated by sampling a finite mixture of topics and then sampling words from each of these topics. The ordering of the words is irrelevant to LDA.

Here we briefly describe LDA. We model each document *w*, from a corpus *D* that contains *N* words as a generative process:

- Choose θ Dir(α).
- For each of the *N* words *w*_*n*_: define a topic *z*_*n*_ Multinomial (θ) and a multinomial probability conditioned on topic *z*_*n*_ (*p*(*w*_*n*_—*z*_*n*_, β).

where Dir is the Dirichlet function, α is a *k*-dimensional vector parameter with α_*i*_ > 0. For *k* topics the probability density of θ is defined as:

(1) $$p({\rm{\theta}}|{\rm{\alpha}})= {{{\rm{\Gamma}}(\sum_{i=1}^k {\rm{\alpha}}_i)}\over{\prod_{i=1}^{k} {\rm{\Gamma}}({\rm{\alpha}}_i)}} {\rm{\theta}}_{1}^{{\rm{\alpha}}_1-1} \ldots {\rm{\theta}}_{k}^{{\rm{\alpha}}_k-1}$$

where Γ is the Gamma function.

Given the auxiliary parameters α and β, the joint distribution of a topic mixture θ, topic *z* and word *w* is defined as:

(2) $$p({\rm{\theta}},z,w | {\rm{\alpha}},{\rm{\beta}}) = p({\rm{\theta}} | {\rm{\alpha}})\prod_{n=1}^N p(z_n | {\rm{\theta}})p(w_n | z_n,{\rm{\beta}}),$$

where *p*(*z*_*n*_|θ) = θ_*i*_ such that $z_n^i = 1$. To obtain the document probability density we can marginalise over θ and *z*.

(3) $$p(w|{\rm{\alpha}},{\rm{\beta}}) = \int p({\rm{\theta}}|{\rm{\alpha}}) \bigg(\prod_{n=1}^N \sum_{z_n}p(z_n | {\rm{\theta}})p(w_n | z_n,{\rm{\beta}})\bigg) d{\rm{\theta}}$$

The corpus probability is then defined as:

(4) $$ p(D|{\rm{\alpha}},{\rm{\beta}}) = \prod_{d=1}^M \int p(w_d|{\rm{\alpha}},{\rm{\beta}})dw_d $$

Efficient parameter estimation is usually done through variational Bayesian methods or Gibbs sampling (Blei, Ng & Jordan, 2003). The complete description of LDA is beyond the scope of this paper, interested readers are referred to Blei, Ng & Jordan (2003).

LDA has been widely used for text modelling and classification. In the context of sentiment analysis several studies tried to extend the original LDA model for polarity classification (Eguchi & Lavrenko, 2006; Mei et al., 2007; Lin & He, 2009). However, these methods focused on adding an additional layer of abstraction (via latent variables) to describe the different sentiments of interest. This is limited to the strong assumptions put into the Bayesian network. In this work we use a deep neural network (explained in the next section) which can model complex relationships among topics that are responsible for generating the documents. This approach requires minimum assumptions about the relationship between topics/documents and sentiments.

### Stacked denoising autoencoders

Stacked AE fall under the umbrella of representative learning using deep neural networks. The goal of an AE is to learn an abstract representation of the data presented at its input. The input can be reconstructed from that representation. Hence the desired output of the AE is the input itself (Bengio, 2009; Bourlard & Kamp, 1988; Japkowicz, Hanson & Gluck, 2000). Hinton & Zemel (1994) defined an AE as ‘a network that uses a set of recognition weights to convert an input vector into a code vector. It then uses a set of generative weights to convert the code vector into an approximate reconstruction of the input vector’.

**Figure 2:**
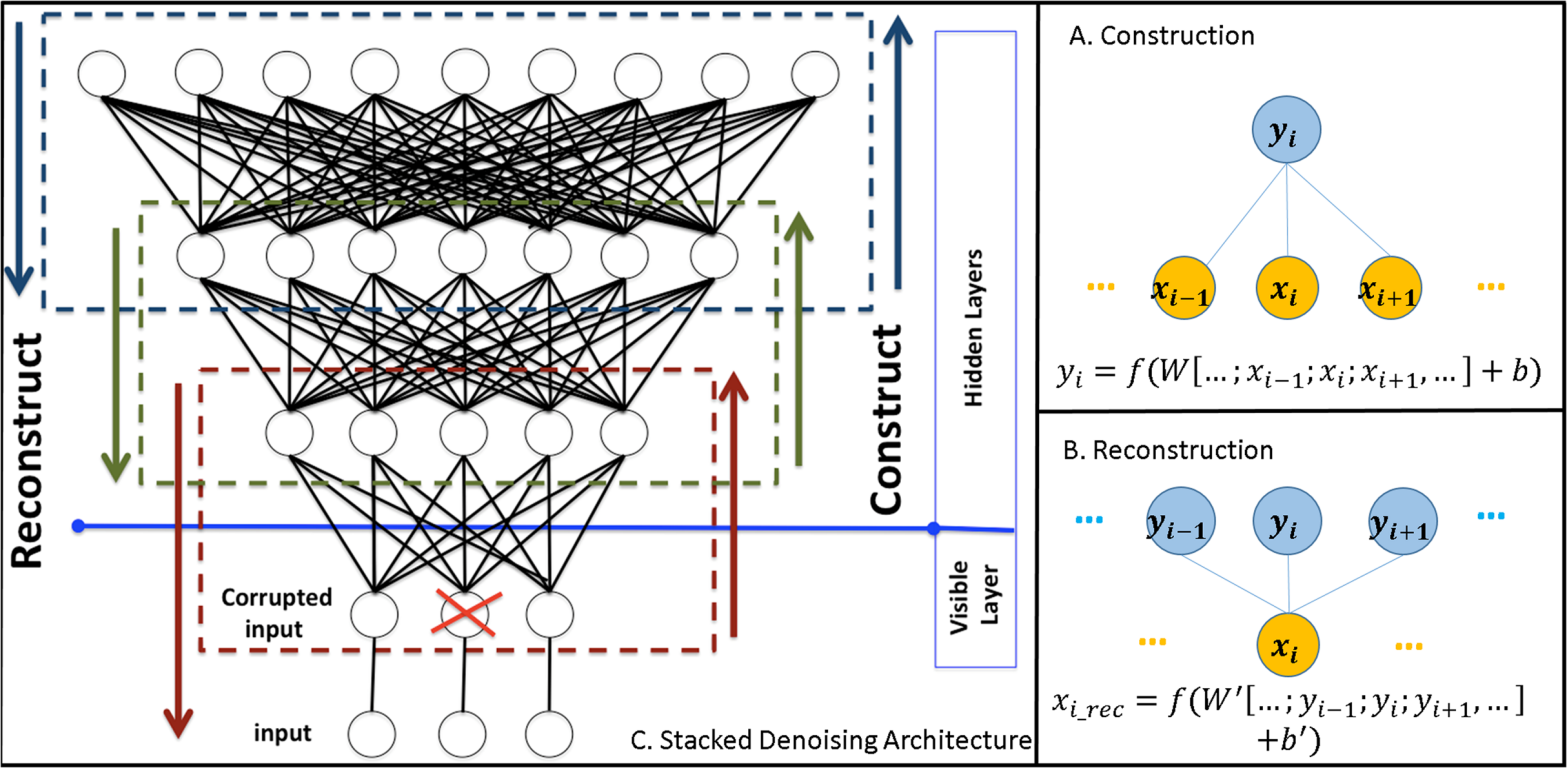
Demonstration of denoising stacked autoencoders. (A) demonstrates the process of constructing an autoencoder. Yellow circles represent an input layer and blue circles represent the hidden layer. (B) demonstrates the reconstruction process of the input from the units in the hidden layer. (C) A stacked denoising autoencoder. Each dashed rectangle represents an autoencoder. *X* represents a connection corrupted by noise.

Assuming we have a network of just two layers: an input (visible) layer of *m* dimensions *x* = (*x*_1_, *x*_2_, …, *x*_*m*_) (e.g. topic modelling features as described in the previous section) and a hidden layer of *n* nodes *y* = (*y*_1_, *y*_2_, …, *y*_*n*_). Each node in the hidden layer is connected to all the nodes in the input layer. In the construction phase we compute the hidden representation:

(5) $$ y=f(Wx+b) $$

where *W* ∈ *R*^*n*^^×^^*m*^ is a weight matrix and *b* is a bias term. Figure 2A demonstrates a simplified example of such a network in the construction phase.

To assess how well the new *n*-dimensional vector *y* represents the *m*-dimensional input *x*, we can reconstruct the input layer from the hidden layer (Fig. 2B):

(6) $$ x_{\rm rec} = W^{T}y+b $$

where *W*^*T*^ is the transpose matrix of *W*.

To train the network (i.e. optimise *W* and *b*) we want to minimise the RE between *x* and *x*_rec_:

(7) $${\rm{RE}} = \sum\limits_{i = 1}^N {||{D^i} - \mathop D\nolimits_{{\rm{rec}}}^i ||_2^2} $$

where *N* is number of m-dimensional input samples, *D*^*i*^ is an input sample that is fed to the network, and *D*^*i*^_rec_ is the reconstructed version using Eq. (6).

In this article we use the denoising variant of an autoencoder (DAE) (Vincent et al., 2010), which corrupts the inputs with added noise in order to enhance the generalisation of the network and hence enhance its representational power. The motivation behind adding this noise factor is to avoid over-fitting, that is the network learns to model only the training samples. Figure 2C demonstrates the corruption process which randomly (using an isotropic Gaussian distributed noise) assigns a weight of 0 to the link between two nodes. DAEs are trained with standard stochastic gradient descent and usually perform significantly better than the standard AE (Vincent et al., 2010).

Deep architectures facilitate the modelling of complicated structures and patterns efficiently (Ngiam et al., 2011; Vincent et al., 2010). Within the framework of DAE the deep network is built by stacking layers of denoising AEs that are trained locally, as explained above. The output/hidden layer of each AE plays the role of the input layer of the deeper network (Vincent et al., 2010) (Fig. 2C).

As suggested by Vincent et al. (2010), SDA are usually used for feature extraction or dimensionality reduction followed by a classifier (e.g. SVM). Alternatively an additional logistic regression layer is added on top of the encoders, which serves as a supervised deep neural network (Hinton, Osindero & Teh, 2006; Hinton & Salakhutdinov, 2006). The parameters of the whole network are then optimised using standard gradient-based methods with the original SDA playing the role of unsupervised pre-training model.

#### Overall reconstruction error

Here we refer to the RE between layers as local reconstruction error. The RE as defined in Eq. (7) between the input data (e.g. topic modelling features) and the reconstructed features becomes a measure of the overall reconstruction error (ORE).

Alain & Bengio (2014) showed that by minimising a particular form of regularised ORE stacked AEs capture the density generating the modelled data. This motivates the use of ORE as a surrogate measure of the ‘goodness’ of representation of an input example by the network. A high ORE suggests poor representation of the input sample while a small ORE is an indication of an accurate representation of the input.

We, AlMoubayed et al. (2016), used ORE as an indication for outlier detection. The novel use of ORE here is as the feature extracted from each SDA model. As each SDA is trained on the output of a topic model associated with one class, the samples of other classes will produce high ORE while samples of the same class will generate low ORE. This results in easily separable (usually linearly) feature space as will be discussed later on. Bengio, Courville & Vincent (2012) and Erhan et al. (2010) demonstrated the reasons why unsupervised representational learning, including SDA, are able to model complex structure in data. The depth of the network, defined by the path between the input features and the output layer allows for efficient modelling of abstract concepts. The hierarchy of nodes in the network works as a distributed information processing system which proved to be beneficial in many application areas (Bengio, Courville & Vincent, 2012). In the next section we will provide further details of how we use this in our approach.

### Classification approach

We take a supervised classification approach here. Data from each class are first transformed from a word/document space to the topics space using the topic modelling approach in “Topic Modeling”. The topic modelling features of data from both polarities are then used to build a SDA model, the result of this process is *C* SDA networks, where *C* is the number of classes (sentiment polarities). The data from all classes is then passed through these networks to obtain *C* OREs. A classifier is trained on the OREs to predict the class.

This process is outlined in Fig. 1. In this example a positive vs. negative polarity prediction is targeted. The input data can come from a wide range of resources (e.g. twitter, blogs, product reviews, structured data). LDA is built separately for the positive and negative polarities (with a preset number of topics). In the next phase two SDA networks are built, with different architectures. All the input data (negative or positive) is then passed through the LDA and SDA phases and for each sample ORE1 (positive) and ORE2 (negative) are calculated to be used by a classifier for final prediction.

The motivation behind this approach can be summarised as following:

- By building a separate SDA model per class the OREs are considered representative of how much a document (i.e. input sample) belongs to either polarity. Hence the two OREs provide easily separable features to the final classifier (Bengio, Courville & Vincent, 2012).
- The use of topic modelling adds an extra layer of abstraction which helps combine different resources of data, handle adaptive and continuously changing input data (e.g. input from social media) and makes the SDA modelling less prone to outliers in the input space.
- The resulted approach requires only two parameters: the number of topics necessary for LDA and the architecture of the two SDAs. In “Results” we analyse carefully the effect of the number of topics on the performance of the whole system. The SDA architecture is a much more difficult parameter to tune and usually depends on skilled network designer.

## Data Sets and Experiments

Here we will focus mainly on sentiment analysis data as an example of a text classification problem. Sentiment analysis covers a wide range of tasks/applications. One of the most common tasks is the analysis of product reviews (such as movies) into positive, negative and neutral (Lin & He, 2009; Maas et al., 2011; Nguyen et al., 2014; Pang, Lee & Vaithyanathan, 2002; Wu & Pao, 2012). Another popular task is the analysis of social media data and more specifically is twitter data analysis (Bravo-Marquez, Mendoza & Poblete, 2013; Saif et al., 2013; Tang et al., 2014a).

In this work we study the application of our method on 10 datasets providing a wide range of data sizes and classification tasks. To unify the analysis under one framework we restrict the tasks to dual polarity problems (i.e. two classes). We limit our selection of datasets to those with manually labelled samples using average ratings by annotators to guarantee the reliability of the accuracy of results reported here.

As an example of additional task is the detection of spam SMS messages. We used this dataset as a evidence of the generalisation of the our framework beyond sentiment analysis.

Table 1 summarises the datasets used including the classification task performed and the number of samples per polarity.

**Table 1:** Datasets.

Dataset ID	Task	No. polarity I	No. polarity II
IMDB	Positive vs. Negative	12,500	12,500
Movie-Rev1	Positive vs. Negative	5,331	5,331
Movie-Rev2	Positive vs. Negative	1,000	1,000
Movie-Sub	Subjective vs. Objective	5,000	5,000
UMICH	Positive vs. Negative	3,091	3,995
MDSD-B	Positive vs. Negative	1,000	1,000
MDSD-D	Positive vs. Negative	1,000	1,000
MDSD-E	Positive vs. Negative	1,000	1,000
MDSD-K	Positive vs. Negative	1,000	1,000
SMS-Spam	Spam vs. Ham	747	4,827

The following is a brief description of these datasets:

- *IMDB Movie Review dataset (IMDB)* (Maas et al., 2011): a 50,000 selected reviews from the internet movie database (IMDB) archive for sentiment analysis. A maximum of 30 reviews are allowed per movie. The dataset contains an equal number of positive and negative samples. Reviews are scored between 1 and 10. A sentiment is positive if IMDB rating ≥7 and negative if <5. Neutral reviews are not included in the dataset.
- *Movie Reviews: sentiment polarity datasets (Movie-Rev1)* (Pang, Lee & Vaithyanathan, 2002): the data was collected from IMDB. Only reviews where the author rating was expressed either with stars or a numerical value are used. Ratings were automatically extracted and converted into positive, negative, and neutral. However, in their original paper the authors limited the analysis to only positive and negative samples. To avoid bias in the reviews a limit of 20 reviews per author was allowed.
- *Movie Reviews: sentiment scale datasets (Movie-Rev2)* (Pang & Lee, 2005): data was also collected from IMDB from four authors. Explicit rating indicators from each document was automatically removed. Annotators are asked to rate the reviews and rank them as positive and negative with ratings averaged per review. Only negative and positive reviews at the extremes are kept.
- *Subjectivity datasets (Movie-Sub)* (Pang & Lee, 2004): the dataset looks at subjective vs. objective phrases. For subjective phrases, the authors collected 5,000 movie review snippets from www.rottentomatoes.com. To obtain objective data, a collection of 5,000 sentences from plot summaries available from IMDB were taken.
- *UMICH SI650—Sentiment Classification (UMICH)* (UMICH, 2011): contains data extracted from social media (blogs) with the goal of classifying the blog posts as positive or negative.
- *Multi-Domain Sentiment Dataset* (Blitzer, Dredze & Pereira, 2007): contains product reviews taken from Amazon.com from 4 product domains: books (MDSD-B), DVDs (MDSD-D), Electronics (MDSD-E) and Kitchen (MDSD-K). Each domain has several thousand reviews. Reviews contain star ratings (1–5 stars) with ratings ≥4 are labelled positive and ≤2 are labelled negative and the rest are discarded. After this filtering a 1,000 positive and 1,000 negative examples per domain are available.
- *SMS Spam Collection Data Set (SMS-Spam)* (Almeida, Hidalgo & Yamakami, 2011): the data was collected from free or free for research sources available online. The SMS messages are manually labelled into ham (real message) and spam (unsolicited messages).

### Experiment setup

As discussed earlier there are two parameters to be set for every dataset: (I) the number of topics used in the topic modelling phase (II) SDA architecture. For each dataset a range of possible numbers of topics was tested between 10 and 300. The architecture of the SDA per polarity per dataset was selected experimentally but all shared the need for an input layer of similar size to the number of topics and two hidden stacked layers of increasing sizes. All units had sigmoid activation functions with the learning rate is set to 0.1 and corruption rate of 30% (normally distributed). The learning algorithm ran for 100 epochs.

**Figure 3:**
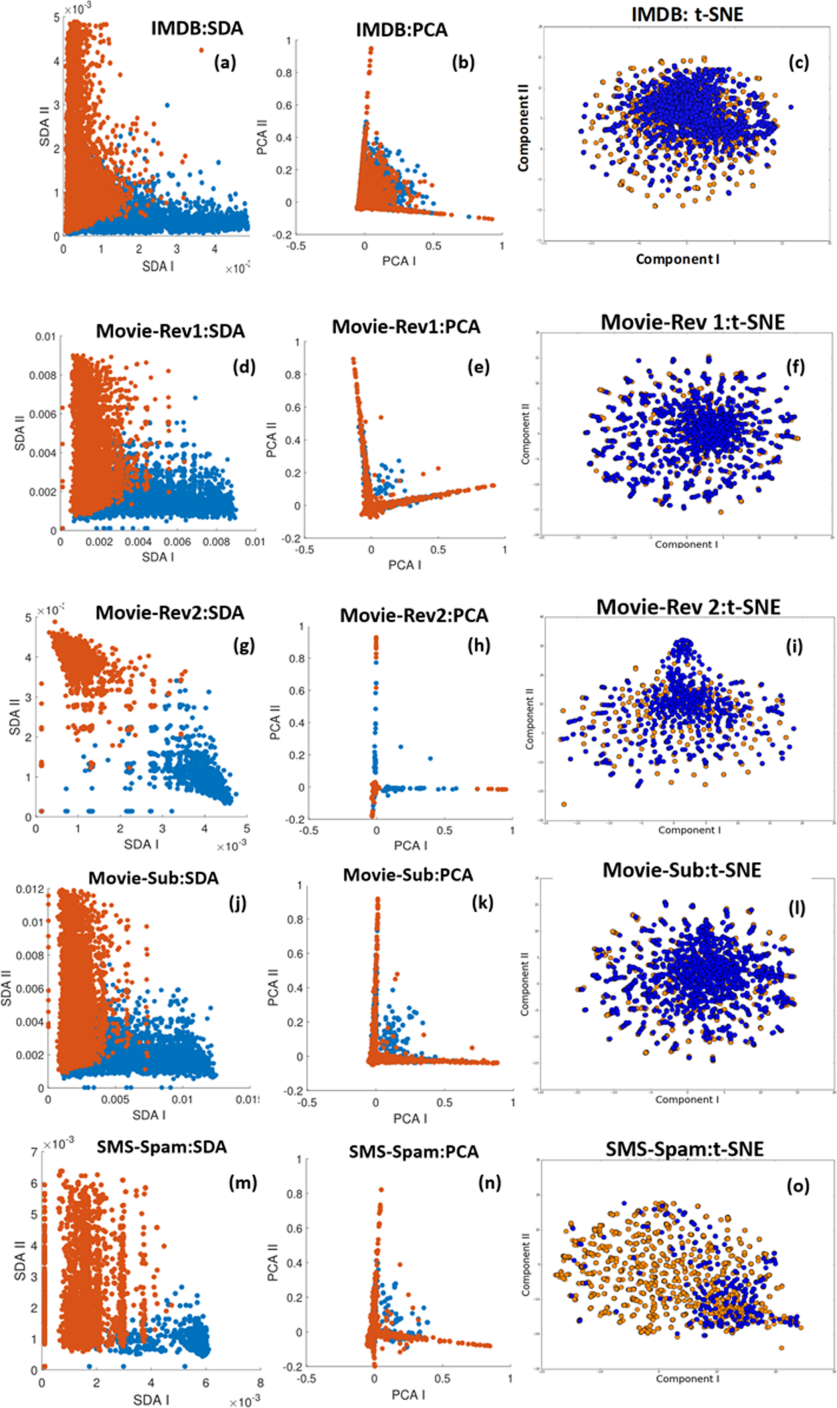
Feature separability of the datasets: IMDB. Movie-Rev1, Movie-Rev2, Movie-Sub, and SMS-Spam. Blue and orange represent the two polarities in the data. (A, D, G, J and M) Demonstrate the ORE generated by first SDA (SDA I) vs. ORE generated by the second SDA (SDA II) (B, E, H, K and N) LDA features projected using PCA (C, F, I, L and O) LDA features projected on a 2D space using the first components of t-SNE.

**Figure 4:**
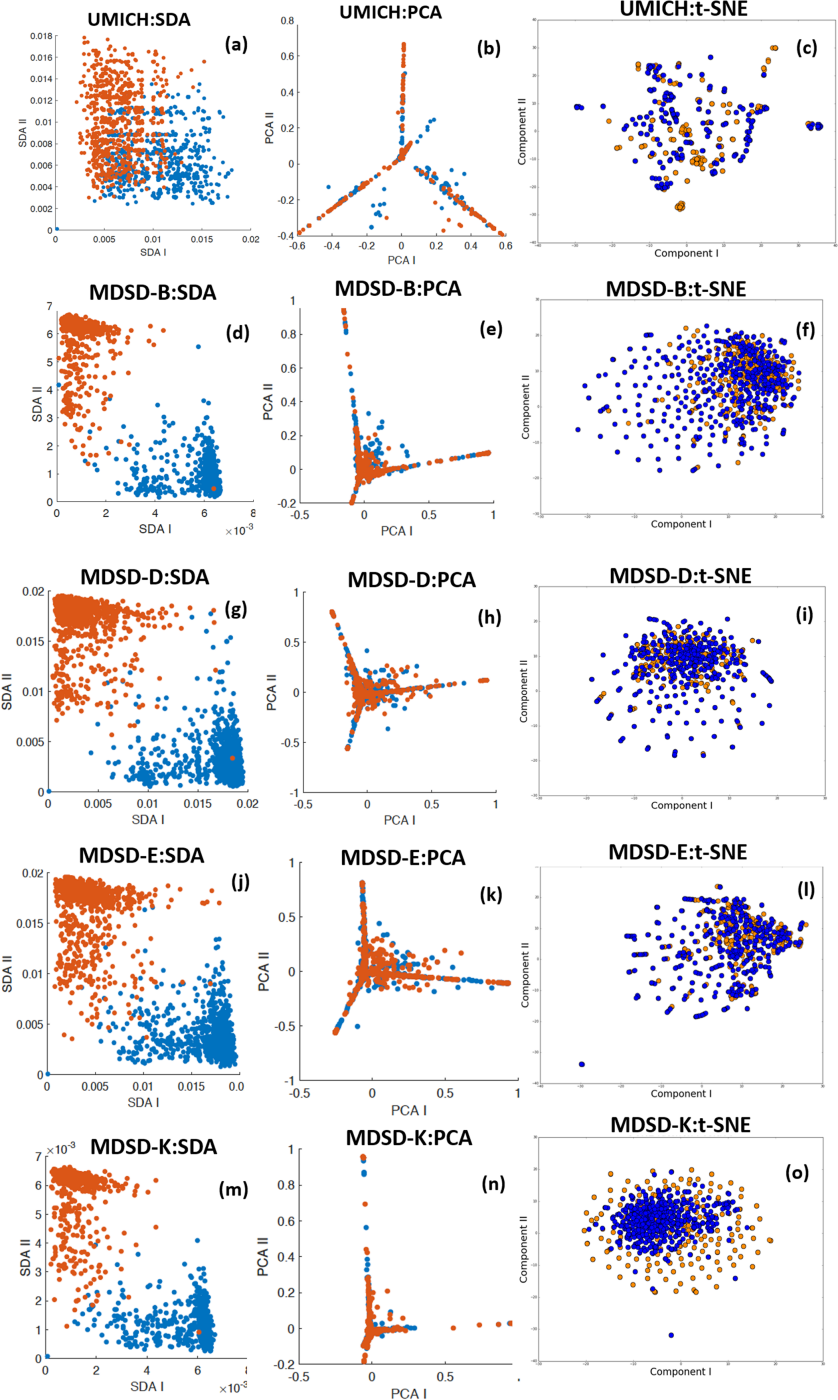
Feature separability of the datasets: UMICH. MDSD-B, MDSD-D, MDSD-E, and MDSD-K. Blue and orange represent the two polarities in the data. (A, D, G, J and M) demonstrate the ORE generated by first SDA (SDA I) vs. ORE generated by the second SDA (SDA II) (B, E, H, K and N) LDA features projected using PCA (C, F, I, L and O) LDA features projected on a 2D space using the first components of t-SNE.

Another important technical aspect is the classifier mentioned in “Classification Approach” to predict the sentiment. Before deciding on the best classification approach to take, we look at the separability of the features generated by the combined LDA+SDA approach compared with the projected topic modelling features on a 2D space using Principle Component Analysis (PCA) or t-Distributed Stochastic Neighbour Embedding (t-SNE). Figures 3 and 4 show with scatter plots the separability of the features for the problems tested here. The figures show from left to right: (I) ORE generated by first SDA (SDA I) vs. ORE generated by the second SDA (SDA II). (II) LDA features projected using PCA (an unsupervised linear method). (III) LDA features projected on a 2D space using the first two components of t-SNE (an unsupervised non-linear method). The results demonstrate the huge benefit of using SDA following the extraction of topic modelling features with LDA. The LDA features are massively overlapped between the classes hence it requires high dimensional features to get a reasonable accuracy. However, by using the SDA generated OREs and with only two features the separability is very high which makes it easier for even a simple linear classifier to achieve high accuracy (more formal discussion will follow in “Discriminability Analysis and Simulations”). To this end we considered three classification methods:

- *ORE based classifier (OBC)*: this is a very simple classifier where the ORE of both SDAs are compared and the sentiment associated with the smaller ORE is marked as the predicted sentiment.
- *Softmax SDA (SoftSDA)*: an additional softmax layer is added on top of the two already trained SDA models. The softmax layer transforms the output into probabilities. The sentiment corresponding to the highest probability is the predicted outcome.
- *Fisher Discriminant Analysis with SDA (FDA+SDA)*: instead of adding a softmax layer, FDA+SDA uses the OREs from both SDAs to train an FDA classifier. FDA is particularly suitable for this task given its linear nature.

To better understand the behaviour of our combined approach we compare it with four other classification methods:

- *Topic Modelling with SVM (TM+SVM)*: each document in the dataset is passed through the two LDA models for both sentiments (e.g. positive and negative). The output of both LDAs (i.e. the probabilities of the document belonging to the topics related to each sentiment) are combined to generate a feature vector. The feature vectors are classified using a support vector machine with a Gaussian kernel.
- *Topic Modelling with Confidence of SVMs (TM+CSVM)*: in this approach the LDA features of sentiment (*S*_1_) are used to build an SVM classifier to discriminate between the two sentiments (*S*_1_, *S*_2_). Similarly another SVM classifier is built based on *S*_2_ LDA features. The final classification output is decided by the SVM classifier with the highest output confidence.
- *Topic Modelling with Logistic Regression (TM+LR)*: this is equivalent to TM+SVM after replacing the SVM classifier by a regularised logistic regression one.
- *Topic Modelling with Confidence of LRs (TM+CLR)*: similar to TM+CSVM, this approach builds a separate regularised logistic regression classifier per LDA model and the confidence in the output is used to make the final decision.

For every dataset we use a 10 fold cross validation approach to evaluate the accuracy of the system with a variable range of the number of topics and the seven classification strategies. “Results” details the results per dataset with comparison with the state-of-the-art of every dataset.

### Baseline methods

To establish baseline and to compare with the state of the art methods, we implemented a list of common methods/approaches in the literature across all the datasets:

- BagOfWords+SVM: bag of words is used for feature extraction and SVM is the classifier.
- Bigram+SVM and Unigram+SVM: bigram/unigrams are used for feature extraction and SVM is used as the classifier.
- LDA+SVM: one LDA models is built for feature extraction using all the training data, completely unsupervised. Number of topics is selected similar to the approach taken in this paper. SVM is then used as the classifier.
- Lexical+SVM: sentiWordNet (Baccianella, Esuli & Sebastiani, 2010) is used to extract features that are then used by an SVM classifier.
- Glove+LSTM: glove English language model as implemented in spaCy (Spacy, 2019) is used in line with a Long-Short Term Memory (LSTM) as a classifier. The optimisation of the model parameters is done independently for each test datasets as in (Hong & Fang, 2015).

SVM is used for most of the base line methods as it in one of the most commonly used classifier for methods that do not use language models. A linear and Gaussian kernels were tested and a choice based on the cross-validation performance was selected. A 10 fold cross validation is used across the board including the baseline and our proposed approaches. The cross validation split is the same for all the methods with the training data used to optimise any parameters for the feature extraction and classification methods. More details about and wider range of comparisons can be found in the cited work per dataset as explained above.

## Results

**Figure 5:**
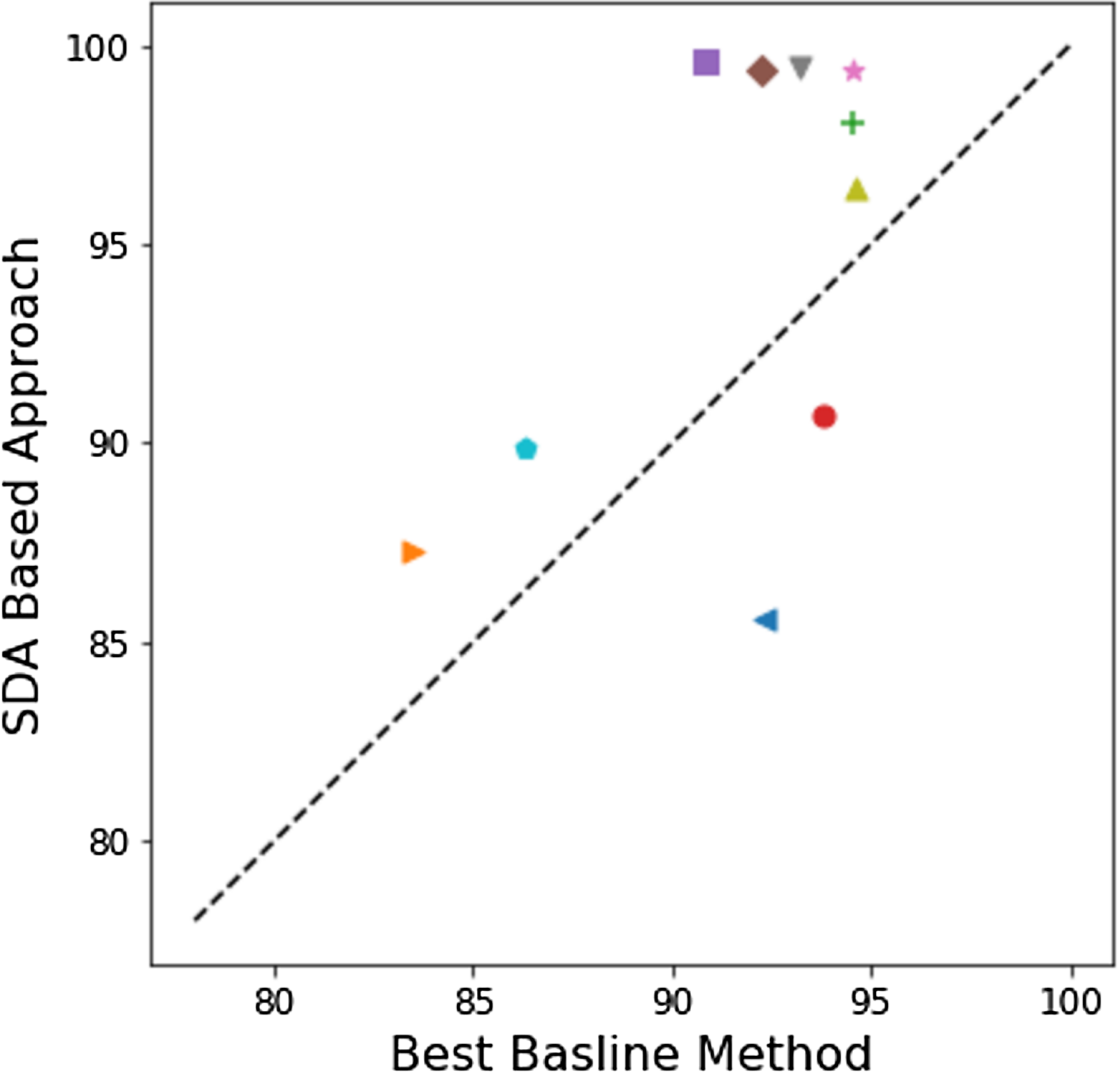
A scatter plot to compare the best in the compared baseline methods from the literature per problem to our approach. If dots are below the line it mean the results in the literature are better otherwise our approach is better.

Figure 5 uses a scatter plot to demonstrate the improvement in performance of our approach to the compared baseline methods in almost every dataset studied here despite the wide range of tasks and challenges offered by these sets. In Fig. 5 as most shapes in are above the equilibrium line, but a *t*-test does not show a significant difference *p* > 0.05. Glove+LSTM seems to be dominant across of the other baseline methods. Removing it from the comparison set, then our SDA base approach is significantly better with *p* < 0.05. This strongly suggest that the proposed approach here is comparable to the much more computationally expensive language modelling based methods.

**Figure 6:**
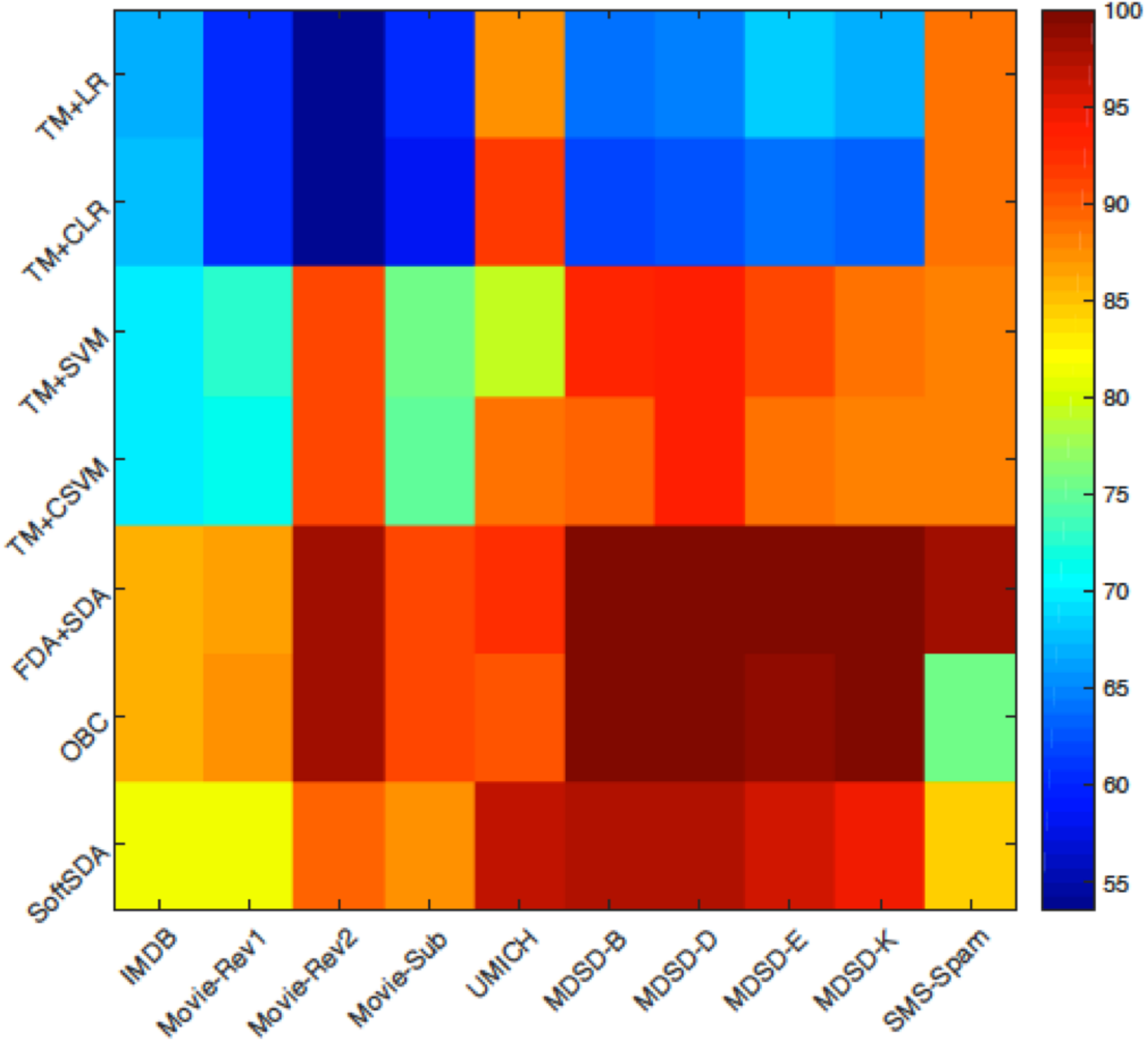
A colour map visualising the performance of the different methods on the ten datasets. The colour scale on the right of the figure clarifies the colour code with dark blue indicating low classification accuracy and dark red reflects high classification accuracy.

Figure 6 summarises the results achieved by all the classification approaches proposed in this study when applied on all the datasets. In the following we discuss these results in more details per dataset and compare these results to the state-of-the-art. In “Discussion” we analyse the reasons behind the advantage of our approach.

### IMDB movie review dataset

**Figure 7:**
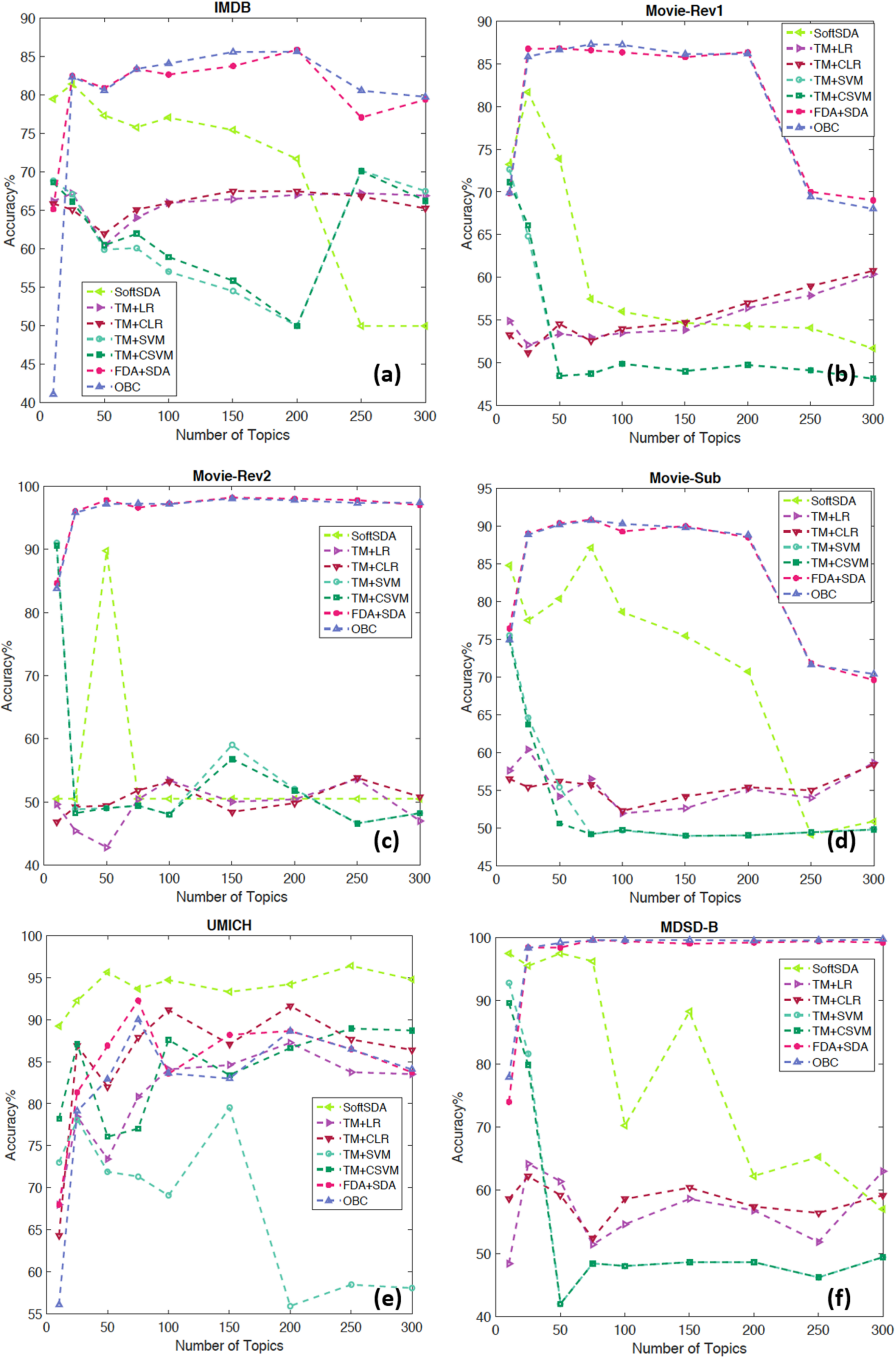
Detailed results showing the change in accuracy with number of topics and different classification schemes for the datasets: (A) IMDB, (B) Movie-Rev1, (C) Movie-Rev2, (D) Movie-Sub, (E) UMICH, and (F) MDSD-B.

Figure 7A details the accuracies per classification scheme which are coded in different colours and shapes. It also illustrates the change of cross validation accuracy with an increased number of topics. The methods vary in performance with FDA+SDA and OBC outperforming the rest and showing consistent results regardless of the number of features. SoftSDA achieves high accuracy but only when using a small number of topics. The classification methods that bypass SDA achieve much lower accuracies even with low number of features.

Maas et al. (2011) compared their method (which uses a probabilistic LDA like method) to other common methods in the literature based on a 10 fold cross validation scheme similar to our validation approach here. Another comparison was reported by Socher et al. (2013) where they compared with common classifiers including Naive Bayesian (NB), SVM, biNB and RNNs. Table 2 summarises these results.

**Table 2:** IMDB results.

Method	Accuracy (%)
BagOfWords+SVM	83.5
Bigram+SVM	89.4
Unigram+SVM	87.5
LDA+SVM	88.6
Lexical+SVM	85.4
Glove+LSTM	92.3
Our approach	85.61

### Movie reviews: sentiment polarity datasets (Movie-Rev1)

The parameter tuning results are presented in Fig. 7B. Similar to IMDB, FDA+SDA and OBC perform better and most consistently than the other methods on Movie-Rev1. It is interesting to notice the drop in accuracy for all those methods that do not use use SDA with the increased number of topics. Table 3 compares our results to those reported in the literature (Pang, Lee & Vaithyanathan, 2002) with OBC outperforming all the other approaches.

**Table 3:** Movie-Rev1 results.

Method	Accuracy (%)
BagOfWords+SVM	75.2
Bigram+SVM	79.1
Unigram+SVM	82.7
LDA+SVM	77.3
Lexical+SVM	72.9
Glove+LSTM	83.5
Our approach	87.28

### Movie reviews: sentiment scale datasets (Movie-Rev2)

FDA+SDA and OBC again outperform the other methods for this dataset (Fig. 7C). However, it is clear that the increased number of topics negatively affected the accuracy of SoftmaxSDA. Table 4 shows the superiority of our approach compared with other methods in the literature which are surveyed in detail by Maas et al. (2011) and Pang & Lee (2004).

**Table 4:** Movie-Rev2 results.

Method	Accuracy (%)
BagOfWords+SVM	86.9
Bigram+SVM	88.1
Unigram+SVM	87.5
LDA+SVM	85.6
Lexical+SVM	86.4
Glove+LSTM	94.5
Our approach	98.05

### Subjectivity datasets (Movie-Sub)

The previous datasets focused on negative vs. positive polarity discrimination. On the other hand Movie-Sub focuses on the task of classifying movie reviews based on their subjectivity. Figure 7D shows the detailed results while Table 5 shows our approach performs similarly to the best in the literature with the same pattern of accuracies repeated even with the change of task.

**Table 5:** Movie-Sub results.

Method	Accuracy (%)
BagOfWords+SVM	90.2
Bigram+SVM	93.8
Unigram+SVM	92.5
LDA+SVM	67.6
Lexical+SVM	88.6
Glove+LSTM	92.5
Our approach	90.72

### UMICH SI650—Sentiment Classification (UMICH)

The data was the core part of a challenge on the machine learning competition website (Kaggle, San Francisco, CA, USA) (UMICH, 2011). Figure 7E shows accuracy as high as 96.4% with SoftmaxSDA and 250 topics, which indicates a very high performance for our approach. OBC and FDA+SDA also show above 85% accuracy with comparable results to TM+CSVM, TM+LR, and TM+CLR. The compared accuracy results are detailed in Table 6.

**Table 6:** UMICH results.

Method	Accuracy (%)
BagOfWords+SVM	92.7
Bigram+SVM	94.6
Unigram+SVM	90.5
LDA+SVM	88.7
Lexical+SVM	91.3
Glove+LSTM	94.5
Our approach	96.4

### Multi-domain sentiment dataset

**Figure 8:**
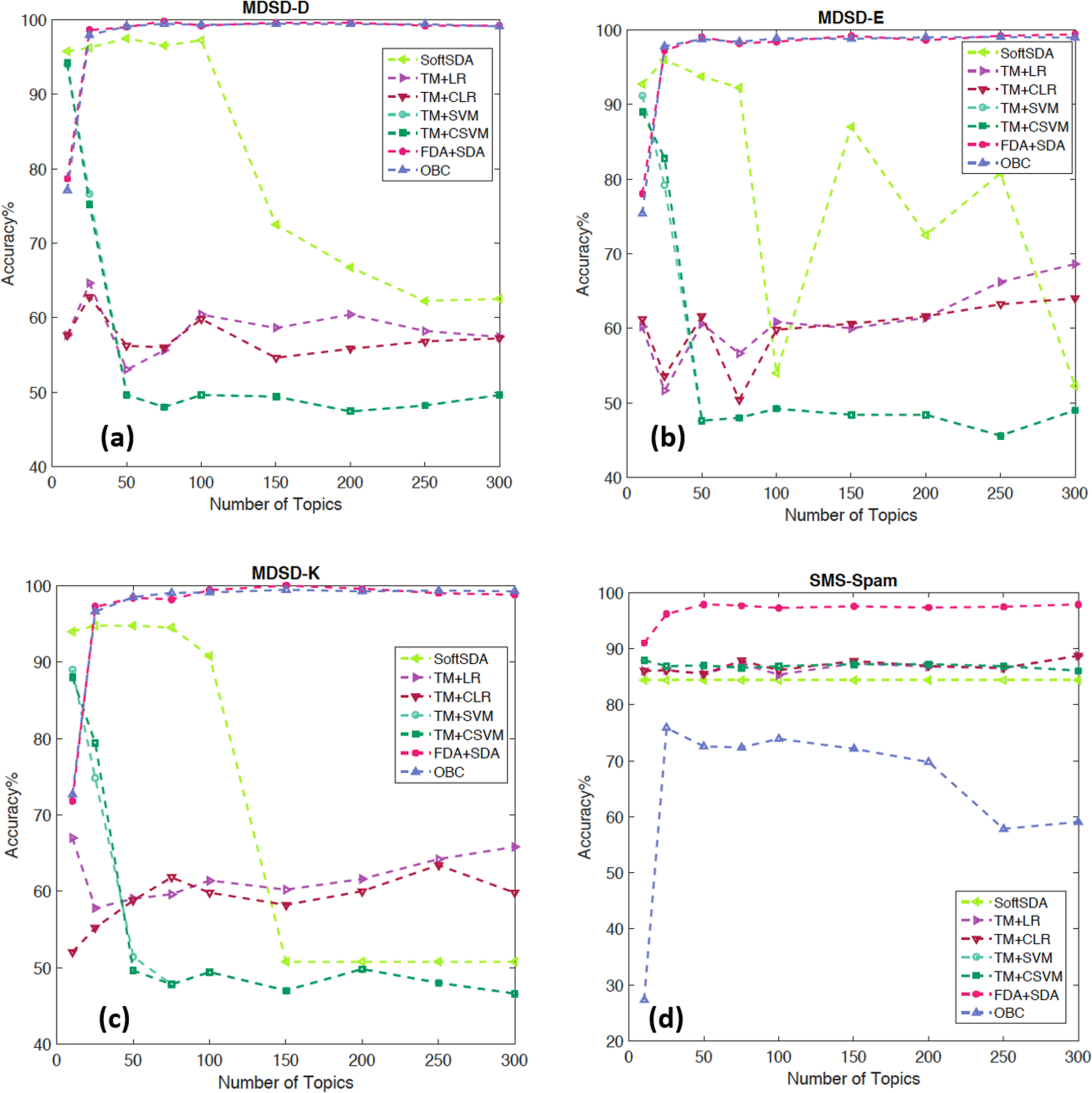
Detailed results showing the change in accuracy with number of topics and different classification schemes for the datasets: (A) MDSD-D, (B) MDSD-E (C) MDSD-K, and (D) SMS-Spam.

Dang, Zhang & Chen (2010) used a lexicon-enhanced method to extract lexical features of different sentiments to boost an SVM classifier using this dataset. A cross-domain approach was used to study the generalisation of features among different domains using these features (Bollegala, Weir & Carroll, 2013). The authors also reported the in-domain sentiment analysis accuracy, which is compared to our approach in Table 7. Finally Li et al. (2010) used a mixture of lexical features that look at the personal/impersonal views to help better separate the features used in polarity classification. The results clearly demonstrates the superiority of our approach compared with the literature and on all the sub datasets. Figures 7F and 8A–8C present the effect of feature size and classification approach on the results of the 4 datasets with FDA+SDA and OBC achieving the best results.

**Table 7:** Multi-domain sentiment results.

Method	MDSD-B (%)	MDSD-D (%)	MDSD-E (%)	MDSD-K (%)
BagOfWords+SVM	77.8	80.9	84.1	84.6
Bigram+SVM	78.5	81.2	83.3	84.8
Unigram+SVM	75.1	80.4	83.4	83.9
LDA+SVM	78.5	81.6	84.2	84.5
Lexical+SVM	78.8	80.7	83.7	84.1
Glove+LSTM	90.8	92.2	94.5	93.2
Our approach	99.6	99.4	99.4	99.45

### SMS spam collection data set (SMS-Spam)

Table 8 compares our approach for the problem of SMS spam filtering with the state-of-the-art on the same dataset. Our approach is on par with the highest accuracy results mentioned in the literature. Figure 8D shows that FDA+SDA scores the highest accuracy with a wide range of feature numbers. Given the data is imbalanced it is important to report complementary performance measures. Our approach has achieved: *F*-score = 92.13%, Precision = 95.47% and Recall = 87.58%.

**Table 8:** SMS spam results.

Method	AUROC
BagOfWords+SVM	85.4
Bigram+SVM	86.3
Unigram+SVM	84.7
LDA+SVM	82.4
Lexical+SVM	84.6
Glove+LSTM	79.8
Our approach	89.9

## Discussion

During the topic modelling phase, LDA groups words within the text into topics with an assigned probability to each word belonging to the topic. A straight forward feature extraction method could be to use a lexical resource to assign each word within the topic with a polarity. A topic is then represented by a weighted average:

(8) $$ T_f = \sum_w{p_w*s_w}, $$

where *p*_*w*_ is the probability of word *w* belonging to topic *T*, $\sum\nolimits_w {p_w} = 1$, and *s*_*w*_ is the polarity assigned to the word by the lexical resource.

**Figure 9:**
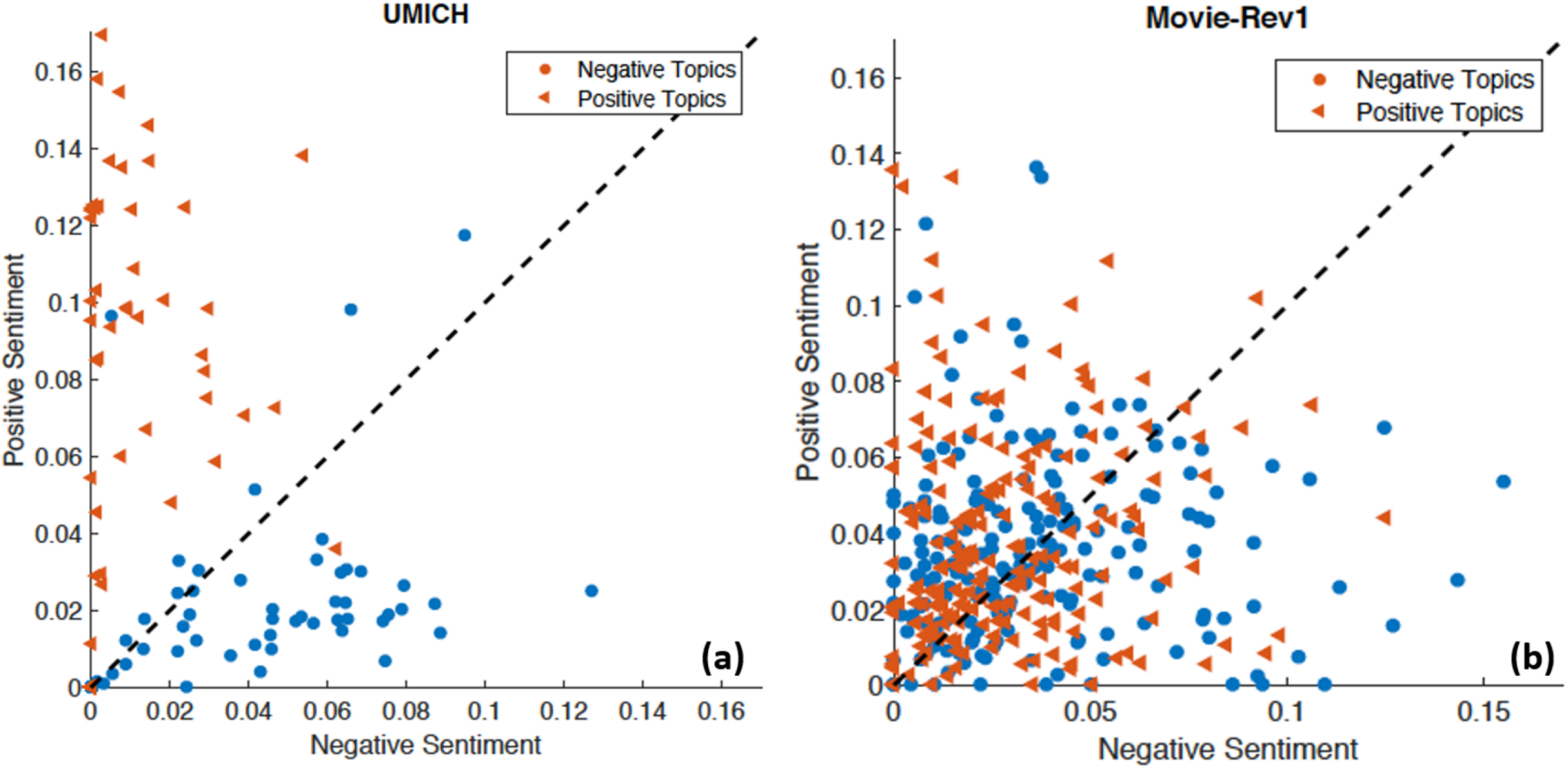
Positive and negative polarity assigned by a lexical resource, SentiWordNet (Baccianella, Esuli & Sebastiani, 2010), to words within a negative and positive topic models. The x-axis represents the negative polarity of a topic, while the y-axis represents the positive polarity. Points over the line means those topics have higher positive polarity, while points under the line carry more negative polarity. (A) Word sentiment from positive and negative topic models for UMICH. (B) The same plot for Movie-Rev1.

Figure 9A shows the positive and negative polarities of positive and negative topics generated when building LDAs using UMICH data. The data clearly show how very well separated the features are which would make it very easy for a linear classifier to perform well. However, in Fig. 9B the same features for Movie-Rev1 data show a high degree of overlap. This exemplifies the importance of using SDA on top of LDA to extract hidden complex relationships among the words within the topics of each sentiment.

The fact that for some datasets the polarity assigned by a lexical resource generates highly separable features, as in Fig. 9, could explain why in some datasets lexical based methods outperformed our approach. However, in the general case where these features are not separable enough the introduction of SDA seems to contribute to the enhanced performance.

In the case of auto-encoders with squared RE, AE is equivalent to PCA (Bengio, Courville & Vincent, 2013). However, as we have shown above PCA is unable to clearly separate the sentiments. Here we used a tied-weight SDA, that is the decoding weight matrix is the inverse of the encode matrix: *W′* = *W*^*T*^, which enforces a non-linear model. The use of denoising in SDA plays the role of regularisation (Bengio, Courville & Vincent, 2013). Regularisation constraints the representation even when it is overcomplete and makes it immune to insensitivity to small random perturbations in the input space. This motivated the increased size of layers with depth, in order to guarantee modelling the data rather than compressing it.

### Discriminability analysis and simulations

**Figure 10:**
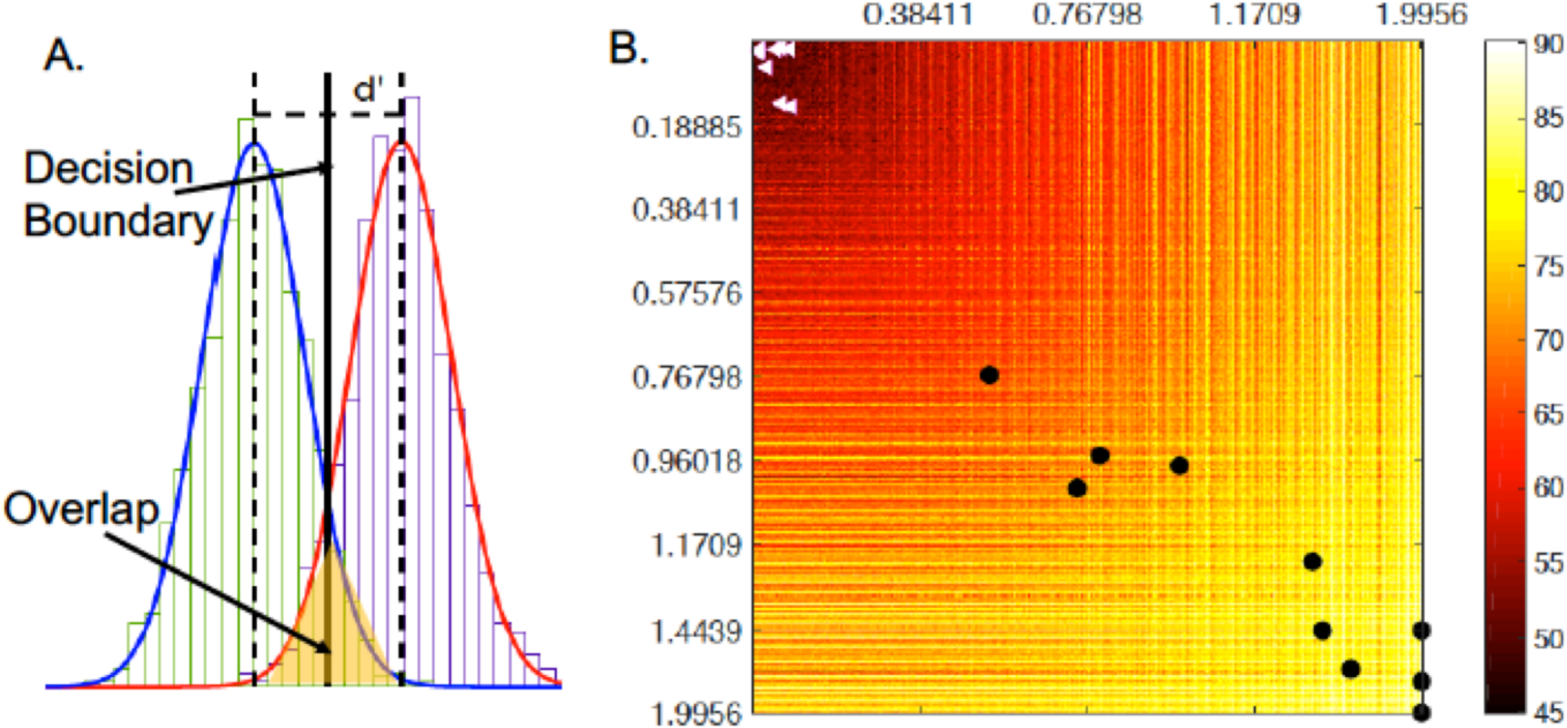
(A) Schematic description of the concept of *d*′; (B) simulations of the effect of changing *d*′ of SDA I and *d*′ of SDA II on the classification accuracy.

In order to understand why the combined approach of topic modelling and SDA works well, we borrow the discriminability index (*d′*) concept from the signal detection theory (Green & Swets, 1966; Neri, 2010). Assume we have two overlapping distributions representing two classes (Fig. 10A). Each distribution is Gaussian with mean μ_*i*_ and variance σ_*i*_. The goal of any learning algorithm is basically to set the decision boundary separating the two distributions in a way that maximises accuracy. Discriminability is made easier by either increasing the separation between the two means (μ_1_, μ_2_) or minimising the spread of the distribution (measured by σ_1_ and σ_2_). *d′* is then defined as the ratio between separation and spread (Macmillan & Creelman, 2004):

(9) $$ d^\prime = {{{\rm{\mu}}_1-{\rm{\mu}}_2}\over{\displaystyle\sqrt{\frac{1}{2}({\rm{\sigma}}_1^2+{\rm{\sigma}}_2^2)}}}. $$

*d′* is a dimensionless statistic with higher values indicating better discriminability, and a higher classification accuracy as a result.

We hypothesise that our approach outperforms the state of the art, due to its ability to increase the discriminability in the output of the two SDAs. This is amplified by the use of class-specific topic model to generate features for the SDAs, which in turn are able to accurately model the data at their input to generate highly discriminable features to be classified using a simple linear classifier as explained above.

To test this hypothesis, we simulate the effect of changing *d*′ on the expected performance of a classifier. The *d′*_*i*_ associated with each SDA output is simulated in the range (0–2). The challenge is to map back from *d′*_*i*_ to μ_*i*_ and σ_*i*_. Each value of *d′*_*i*_ can be generated by an infinite combinations of μ_*i*_ and σ_*i*_. To overcome this problem, we use a Monte Carlo approach to model the joint distribution *p*(*d′*, μ, σ). It is now possible to obtain acceptable values for μ_*i*_, σ_*i*_ given the simulated *d′*_*i*_ value. Details of similar approach can be found in (Neri, 2010).

To calculate the classification accuracy, for each simulated point (*d′*_1_, *d′*_2_) a synthetic data of 1,000 samples is generated from a two-component two dimensional Gaussian mixture distribution (GMM) with mean (μ_1_, μ_2_), a diagonal covariance matrix (σ_1_, σ_2_), and equal mixing coefficients of 0.5. The generated samples are classified using a FDA classifier. The process is repeated 100 times for each point in the simulation space and the average values are reported in Fig. 10B. In the figure a brighter colour indicate higher classification accuracy. It is clear with the increase of *d′* on either or both axes results in higher classification accuracy. The black dots show (*d′*_1_, *d′*_2_) of the output of SDA I and SDA II for all the datasets presented above. The white dots are those produced from the output of the topic modelling without SDA. It is very clear the effect SDA has on the discriminability of the data and hence the accuracy of the overall classifier.

These finding are very important in demonstrating the effect SDA has when combined with topic modelling in the approach described above. It suggests that LDA trained on data from one class helps suppressing the other class(s), SDA then is able to model the inter-dependencies in a way that increases discriminability. This suggest that this approach can be used in other areas beyond sentiment analysis and for larger number of classes.

## Conclusion

This work presented a novel framework for text classification that combines topic modelling with deep stacked AE to generate highly separable features that are then easily classified using a simple linear classifier. The approach transforms the sentiment analysis problem from the space of words (in approaches such as bag of words, and lexical sentiment corpora) to the topics space. This is especially useful as it incorporates the context information within the mixture model of topics (using LDA). To model the class-specific information SDA plays the role of finding structural correlations among topics without any strong assumption on the model. This combination of feature extraction methods results in a semi-automatic approach with minimum feature engineering and number of parameters to tune.

To demonstrate the effectiveness of our approach we used 10 benchmark datasets for various tasks in sentiment analysis and a wide range of data size and class bias. Tasks included negative/positive product and movie reviews, subjectivity of movie reviews, and spam filtering. As presented in the previous section our approach achieves significantly higher accuracies than the best reported in the literature for most of the tested datasets. This and the fact that it requires very little feature engineering makes the approach very attractive for various applications in many domains.

LDA allows for adaptive learning (a feature of Gibbs sampling and variational Bayesian methods) which is a very useful feature for Big Data and streaming applications. SDAs can also be trained in an online manner making the whole system adaptive especially in areas such as micro-blogging and social media.

The work presented here is designed to provide a framework for text classification tasks. Every component in this framework could be replaced by another method. LDA could for example be replaced by TSM or JST. SDA could be replaced by other representational learning algorithms including Restrictive Boltzmann Machines (RBM) or RNNs (RNN). It can also be easily extended to multiple class problems using one-vs-one or one-vs-all methods.

The discriminability analysis presented here provides explanation of why SDA and LDA work well within the presented framework. The analysis further support the claims made in this paper that SDA is able to model the complex structure of class-specific topics and separate them to achieve high classification accuracy.

## Supplemental Information

10.7717/peerj-cs.252/supp-1 Supplemental Information 1 Code of topic modelling and stacked denoising autoencoder. The zip file contains two main folders one for the topic modelling and the other for Stacked Denoising Autoencoder (SDA). SDA is written based on theano.

## Additional Information and Declarations

### Data Availability

The following information was supplied regarding data availability: Code used to generate the topic modelling data is available as in the Supplemental File. The data is publicly available at the following locations: IMDB Movie Review dataset (IMDB): http://ai.stanford.edu/~amaas/data/sentiment/. Movie Reviews: Sentiment polarity datasets (Movie-Rev1): https://www.kaggle.com/nltkdata/sentence-polarity. Movie Reviews: Sentiment scale datasets (Movie-Rev2): https://www.kaggle.com/nltkdata/movie-review. Subjectivity datasets (Movie-Sub): https://www.kaggle.com/nltkdata/subjectivity. UMICH SI650—Sentiment Classification (UMICH): https://www.kaggle.com/seesea0203/umich-si650-nlp. SMS Spam Collection Data Set (SMS-Spam): https://www.kaggle.com/uciml/sms-spam-collection-dataset/discussion/83197. MDSD is available (Multi-Domain Sentiment Dataset (MDSD) at: https://www.cs.jhu.edu/~mdredze/datasets/sentiment/index2.html.
